# Boot Camp: A Randomized Cross-Over Trial of Intensive Upper-Limb Rehabilitation After Chronic Stroke

**DOI:** 10.1177/15459683251348199

**Published:** 2025-07-08

**Authors:** Brenton Hordacre, Jeric Uy, Saran Chamberlain, Ines Serrada, Ashraf N. H. Gerges, Susan Hillier

**Affiliations:** 1 Innovation, IMPlementation and Clinical Translation (IIMPACT) in Health, Allied Health and Human Performance, University of South Australia, Adelaide, SA, Australia

**Keywords:** neurological rehabilitation, stroke rehabilitation, physical and rehabilitation medicine, physiotherapy, treatment outcome, duration of therapy

## Abstract

**Background:**

Stroke recovery is often incomplete. There is a need to robustly evaluate evidence for intensive stroke rehabilitation.

**Objective:**

Investigate feasibility, safety, and preliminary evidence of effectiveness for Boot Camp; a pragmatic, intensive, group-based, 5-week upper-limb rehabilitation program for individuals with chronic stroke.

**Methods:**

A pragmatic randomized cross-over trial allocated individuals with chronic stroke to Boot Camp or usual care. Boot Camp delivered 90 hours of upper-limb rehabilitation in a group setting over 5 weeks. Feasibility was evaluated with recruitment rates, adherence, program completion, acceptability, and safety. Clinical characteristics including time since stroke, age, and corticospinal tract integrity were documented. The primary outcome measure was the Fugl-Meyer Upper Extremity (FM-UE). Secondary measures assessed upper-limb activity, quality of life, and self-efficacy. Interviews at the completion of Boot Camp provided insights into participant experiences.

**Results:**

Thirty-nine individuals consented, with 38 completing the program (22 male, age 61.5 ± 14.8 years, 2.8 ± 3.4 years since stroke). Feasibility criteria for recruitment, program completion, acceptability, and safety were met, but not adherence to full therapy amount. Boot Camp led to large gains in the FM-UE scores (10.2 ± 4.8, *P* < .001), upper-limb activity (7.3 ± 8.7, *P* < .001), quality of life (9.3 ± 22.1, *P* = .012), and self-efficacy (6.1 ± 13.5, *P* = .023). Participants reported themes of intensity matters, variety generally works, peer support, goals are motivating.

**Conclusion:**

Boot Camp was feasible, safe, and led to large and meaningful gains in upper-limb outcomes in individuals with chronic stroke.

## Introduction

Stroke remains a leading cause of adult disability, contributing 116 million disability adjusted life years lost annually.^1^ For many, upper-limb recovery is incomplete,^2,3^ leading to persistent disability that affects self-care and reduces quality of life.^4,5^ Individuals with stroke identify upper-limb rehabilitation as a priority,^6^ yet services are limited. Less than 8 min/day of upper-limb rehabilitation is provided early after stroke,^7^ which is unlikely to maximize recovery during a critical phase of heightened brain plasticity.^8^ Further, observation of inpatient and outpatient rehabilitation found 51% of sessions included upper-limb training with an average of 32 repetitions,^9^ far fewer than the 10 000 repetitions shown to induce neural changes.^10^ In the community, services are often limited to 1 to 2 sessions of 20 to 30 minutes/week.^11^ With such little training, it is perhaps unsurprising data points toward an early recovery plateau within months of stroke.^12-14^

Large amounts of rehabilitation are associated with meaningful upper-limb gains. For example, 35 individuals with chronic stroke completed 300 hours of rehabilitation over 12 weeks, achieving improvements up to 11 points on the Fugl-Meyer Upper Extremity (FM-UE),^15^ far exceeding the clinical important difference of 5.25.^16^ A UK clinical service, delivering 90 hours of intensive, timetabled, upper-limb therapy over 3 weeks reported similar gains. Data from 224 patients (median 18 months post-stroke) reported gains in activity (Action Research Arm Test [ARAT]; 6 points) and impairment (FM-UE; 6 points) that were maintained beyond program completion.^17^ The magnitude of improvement is impressive compared to past trials. For example, 5 to 30 hours of rehabilitation has achieved limited improvement (eg, 2-3 point FM-UE gain).^18-20^ The results therefore appear compelling; intensive rehabilitation leads to significant changes in clinical outcomes for individuals with stroke.

While impressive, there is a need to test ways of delivering such intensive stroke rehabilitation in a randomized controlled trial to then influence clinical guidelines. Furthermore, understanding whether benefit is more pronounced for a subgroup of individuals with stroke might help guide clinical practice or inform stratification and inclusion criteria in future trial design.^21^ Therefore, the aim of this study was to investigate feasibility, safety, and preliminary evidence of effectiveness for Boot Camp; a pragmatic, intensive, group-based, 5-week upper-limb rehabilitation program in individuals with chronic stroke. A recovery biomarker, recommended by international consensus,^21^ for corticospinal tract integrity, was evaluated to understand response to intensive training. We hypothesized that intensive upper-limb rehabilitation would be feasible, safe, and lead to clinically meaningful gains in upper-limb outcomes immediately following the intervention. In this study, “intensity” of training referred specifically to the increased amount of therapy delivered. Finally, we hypothesized that those with an intact corticospinal tract would show greater improvement.

## Methods

### Design

A randomized cross-over study was conducted to evaluate Boot Camp compared to usual care. A cross-over design was selected to ensure all participants were afforded the opportunity to participate in Boot Camp, while ensuring rigor and minimizing risk of confounders with patients acting as their own control. Upon enrolment, individuals were randomly allocated via a computerized random number generator to Boot Camp or usual care first, with sequences held by an investigator not involved in data collection or intervention delivery. After the first study arm, there was a minimum of 5 weeks before swapping to the alternative arm. Outcome measures were obtained at multiple timepoints (pre Boot Camp, post Boot Camp, pre control, post control) by an unblinded assessor not involved in intervention delivery. Although the protocol included a planned 3-month follow-up, this was not conducted due to feasibility constraints and the cross-over timing; as such, only immediate post-intervention outcomes are reported in this manuscript. Data collection occurred at the University of South Australia (UniSA) Health and Medical Clinic from July 2022 to December 2023. This study was pre-registered (July 2022, Open Science Framework 10.17605/OSF.IO/DAURJ; ACTRN12623000452695). This study had ethics approval from the University of South Australia Human Research Ethics Committee and was reported in accordance with the CONSORT extension for randomized cross over trials.^22^

### Participants

Individuals were eligible for inclusion if they were living in the community with chronic stroke (minimum 6 months since stroke), aged ≥18 years, discharged from rehabilitation (inpatient and outpatient public service), and had upper-limb motor impairment (FM-UE <60/66, allowing potential for a minimal clinically important difference [MCID] improvement). Potential participants were excluded if they had painful shoulder limiting ability to undertake rehabilitation, unstable medical conditions, or inability to provide informed consent. Recruitment occurred through social media, private neurorehabilitation clinics, and clinical networks (eg, Stroke Foundation). An online advertisement was placed on the UniSA Research Volunteers website. A power calculation was performed based on data from a similar clinical service in the UK.^17^ For FM-UE, an allowable difference of 7.3 points, pooled standard deviation of 15.7, power of 90% and *P* < .05 would require 25 individuals. This estimate was conservatively adjusted to 35 to allow for potential drop-out or exploratory sub-group analyses.

### Intervention

Boot Camp was an upper-limb sensorimotor rehabilitation program delivering 90 hours total therapy in a group setting (3-4 participants, 1 therapist). Different therapists led the program on designated days, with schedules consistent each week throughout data collection. Individuals with stroke attended the metropolitan clinic 5 days per week, for 5 weeks. Each week included 18 hours of training delivered as 13 hours in clinic (3 hours on Monday, Wednesday, and Thursday; 2 hours on Tuesday and Friday) and a commitment to 5 hours of home exercises (1 hours per day). Programs were tailored to each individual and aimed to address sensorimotor impairments, improve activity and address individual rehabilitation goals. Prior to Boot Camp, impairments, activity, and rehabilitation goals were documented. Although programs were individualized, Boot Camp consisted of key ingredients of repetitive and intensive training. Training intensity and progression were guided by clinical reasoning, with therapists adjusting task difficulty, complexity, support, or duration. Therapy could include strength and flexibility training, practice of activities of daily living, coordination, sensory retraining, and/or gross and fine motor tasks. Therapy was delivered via traditional movement-based programs, through rehabilitative technology (tablet-based gaming devices, robotics, and virtual reality), and group exercises. Home exercises were delivered via the graded repetitive arm supplementary program,^23^ or pre-recorded rehabilitation videos available via YouTube, with adherence monitored using treatment diaries.

### Control

The control intervention consisted of 5 weeks of usual care, which could include no therapy or privately funded community rehabilitation. Individuals with stroke documented therapy type and duration (if any).

### Outcome Measures

Age, sex, time since stroke, and side affected (paretic side) were documented on admission. Transcranial magnetic stimulation determined the presence (or absence) of a motor evoked potential (MEP) to indicate functional integrity of the ipsilesional corticospinal tract. MEP status protocol was similar to our previous work.^24^ Briefly, participants were screened for brain stimulation safety.^25^ Single, monophasic, pulses were delivered to the ipsilesional hemisphere at 0.2 Hz ± 10% using a 70 mm figure-eight magnetic coil to induce a posterior-anterior current across the hand motor cortex (Neuro-MS Monophasic, Neurosoft Ltd. Ivanova, Russia). Stimulation intensity and coil position were systematically adjusted in an attempt to evoke a consistent MEP. If required, intensity was increased to 100% stimulator output, and if possible, participants were asked to perform a voluntary contraction. Surface electromyography was used to identify MEPs. Electrodes were positioned in belly-tendon montage over the first dorsal interosseous of the paretic hand (22 mm × 34 mm, FIAB, Florence, Italy). Participants were considered MEP+ where >5/10 MEPs (amplitude >50 μV, 15-30 ms window after stimulation) could be observed. Where a consistent MEP was not observed, even at 100% stimulator output with voluntary contraction, the participant was considered MEP−.

Feasibility was evaluated against the following criteria and thresholds: (1) >50% of eligible participants consent to participate, (2) >80% of participants achieve 18 hours of therapy a week (90 hours over 5 weeks), (3) >80% of participants complete 5 weeks of Boot Camp, (4) >80% of participants report the program was acceptable and beneficial (Likert scales ≥4/5), and (5) no serious adverse events. Trial logs documented recruitment rates and therapy diaries recorded duration and sessions completed in Boot Camp. Likert scale questionnaires completed at the end of Boot Camp assessed whether individuals found the program useful, enjoyable, would do again, recommended, beneficial, and friends/family noticed improvements (scale 1-5, 1 = strongly disagree, 5 = strongly agree). Safety was monitored throughout the program. Serious adverse events were defined as those requiring hospitalization. Treatment-emergent adverse events were defined as any event not present prior to initiation of the treatment, or any event already present that worsens in either intensity or frequency following initiation of treatment, and that were clinically attributable to participation in the program.

The primary effectiveness measure was the FM-UE, with secondary measures of ARAT, Stroke Specific Quality of Life scale (SSQoL), Stroke Self-Efficacy Questionnaire (SSEQ), and EuroQol-5 Dimension 5 level (EQ-5D-5L). All FM-UE and ARAT assessments were performed by the same experienced and trained physiotherapists. The FM-UE is a reliable and valid assessment of motor impairment, comprising 33 items (scored 0-66, higher indicates less impairment).^26-28^ The tool assesses movement at the shoulder, elbow, wrist, and hand, and includes coordination, reflexes, range of motion, and strength. Each item is scored in a 3-point ordinal scale. The MCID for the FM-UE is 5.25 points.^16^ The ARAT is a valid and reliable measure of upper-limb function.^29,30^ It assesses 19 items across 4 subscales of grasp, grip, pinch, and gross movement of the upper-limb. Each item is scored on a 4-point ordinal scale. Scores are from 0 to 57 (higher indicates greater function). The MCID is 5.7 points.^31^ The SSQoL measures quality of life across 12 domains (49 items; scores 49-245, higher indicates better quality of life). The SSEQ is a 13-item, self-reported scale of self-efficacy (scores 0-130, higher indicates greater self-efficacy). The MCID is 3.3-3.7 points.^32^ Finally, the EQ-5D-5L is a measure of health-related quality of life. Health dimensions of mobility, self-care, usual activities, pain/discomfort, and anxiety/depression were self-reported on a 5-point scale ranging from “no problem” to “extreme problems/unable.” An EQ-5D-5L score was calculated using Australian preference weights.^33^ The Visual Analog Scale (EQ-VAS) measured self-reported health from 0 (worst health imaginable) to 100 (best health imaginable).

### Interview

After completion of Boot Camp, participants were invited for a semi-structured interview to gain further insight into their experiences. Interviews were conducted by a person (SC) with lived experience of stroke (credibility), familiar with stroke research, and not involved in trial conduct (rigor). Scripts were developed collaboratively by the research team (Appendix 1) and interviews occurred either face-to-face or online and were recorded and transcribed verbatim.

### Quantitative Analysis

Analyses were performed using Statistical Package for Social Sciences (SPSS) software (IBM Corp., Released 2016, IBM SPSS Statistics for Windows, Version 24.0, Armonk, NY, USA) with significance level set at *P* < .05, using an intention-to-treat approach. Feasibility was evaluated descriptively against pre-defined criteria and thresholds. A linear mixed model for each outcome measure (FM-UE, ARAT, SSQoL, SSEQ, EQ-5D-5L, and EQ-VAS) evaluated effectiveness. Given the cross-over design, within-subject comparisons were made between each participant’s Boot Camp and usual care periods. Assumptions of linear mixed models (linearity of explanatory variables, normality of residuals (*Q–Q* plot), homoscedasticity, and independence of residuals) were confirmed. Each model had participant as a random factor and fixed factors of Group (Boot Camp, control), Timepoint (pre, post), and Group × Timepoint interaction. Co-variates were evaluated to determine the model producing the lowest Bayesian Information Criterion or prevented overfitting of the data. Co-variates included age, sex, time since stroke, side affected, MEP status, and randomization order. Sensitivity analyses were conducted to determine whether amount of rehabilitation influenced clinical effectiveness. Independent *t*-tests compared gain in each clinical measure (FM-UE, ARAT, SSQoL, SSEQ, and EQ-5D-5L) after Boot Camp between those completing the full program and those missing a session. Further, a correlation was performed between gain in each clinical measure and number of completed sessions. Carry-over effects of Boot Camp potentially limiting opportunity for improvement during usual care were explored by re-analysis of clinical outcomes excluding those allocated to usual care second.

### Qualitative Analysis

Transcripts from interviews were uploaded to NVIVO for coding. Line by line coding was conducted first, followed by clustering of codes into categories. Themes were initially coded (SC), with a second coder (SH) confirming themes (confirmability). These were discussed by the authorship team to establish they reflected group observations during the trial (triangulation). A reflexive process, including member checking, was applied throughout to ensure research bias was managed during data interpretation.^34^

## Results

### Demographics and Clinical Characteristics

**Figure 1.**
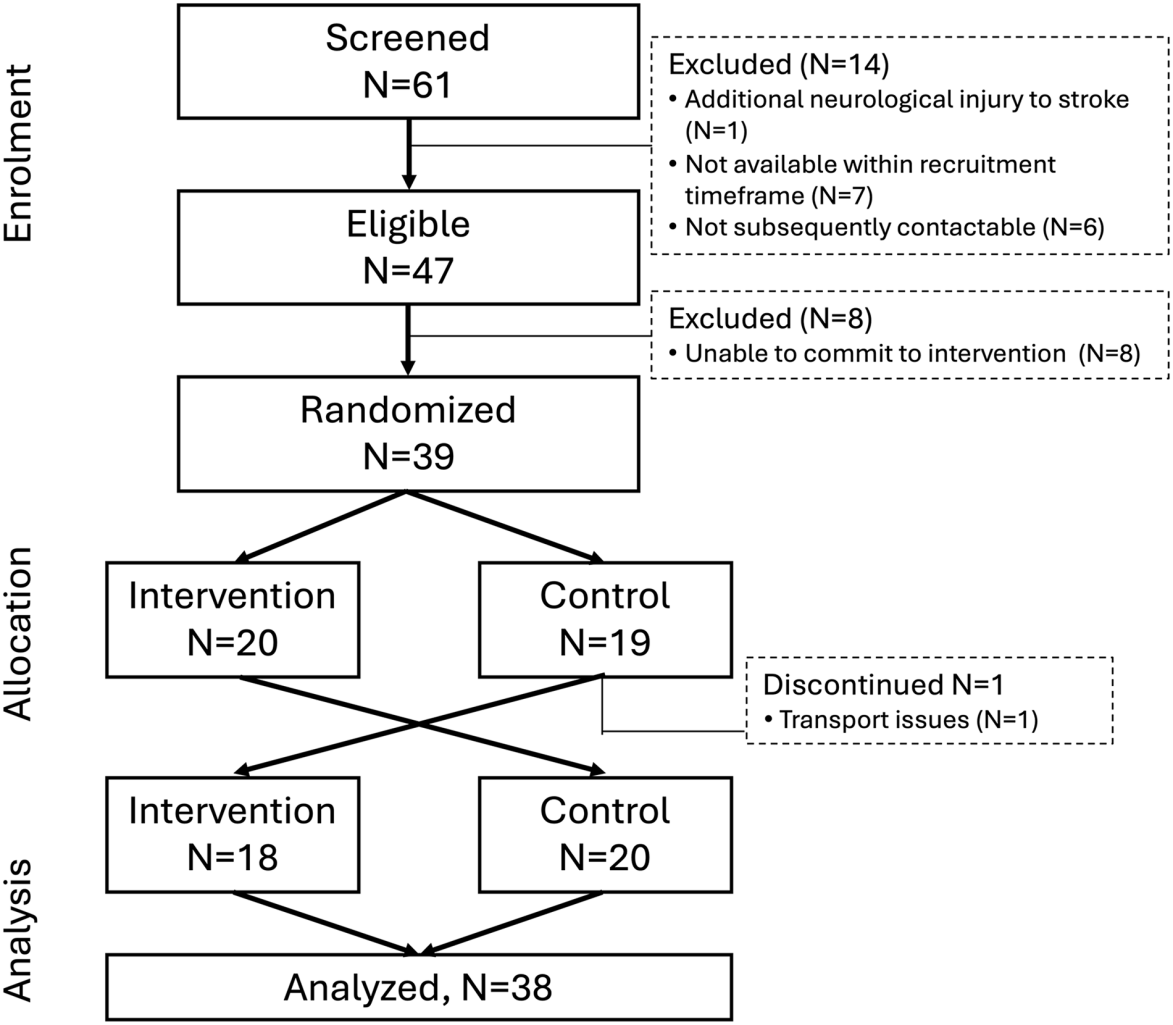
Participant flow chart.

Thirty-nine individuals with stroke were randomized, with 38 completing the study (Figure 1, Table 1, Appendix 2 for de-identified individual data). Usual care was 1.3 ± 0.8 (min–max = 0-3) treatment sessions of 39.6 ± 18.4 minutes duration (min–max = 0-90), for 58.2 ± 43.3 minutes of therapy each week (or 4.7 ± 3.6 h over 5 weeks). Thirty-four participants had active usual care with a therapist, with 19 of those receiving Boot Camp first and 14 control first.

**Table 1.** Demographics and Clinical Characteristics of Individuals With Stroke on Admission.

Variable	Data (n = 38)
Age (years, mean ± SD, min–max)	61.5 ± 14.8, (21-86)
Sex (n (%) male)	22 (58%)
Side affected (n (%) left)	24 (63%)
Time since stroke (years, mean ± SD, min–max)	2.8 ± 3.4, (0.5-17.5)
MEP status (n (%))	24 (63%) MEP+
	12 (32%) MEP−
	2 (5%) unsafe
FM-UE (mean ± SD, min–max)	33.3 ± 18.3, (2-59)
ARAT (mean ± SD, min–max)	24.8 ± 22.2, (0-57)
SSQoL (mean ± SD, min–max)	155.5 ± 41.1, (79-236)
SSEQ (mean ± SD, min–max)	86.9 ± 26.7, (28-126)

Abbreviations: ARAT, Action Research Arm Test; FM-UE, Fugl-Meyer Upper Extremity; MEP, motor evoked potential; SSEQ, Stroke Self-Efficacy Questionnaire; SSQoL, Stroke Specific Quality of Life, SD, standard deviation.

“Unsafe” for MEP status indicates the participant did not pass the brain stimulation safety screening.

### Feasibility and Safety

**Figure 2.**
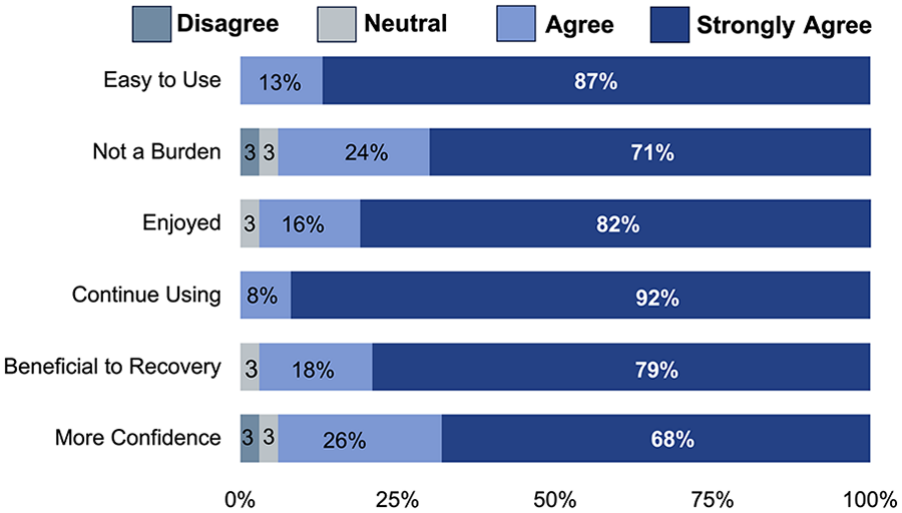
Acceptability scores. *Note.* There were no responses for strongly disagree.

Of 47 individuals eligible, 83% consented, with 97% of those participating in the study. One withdrew after consenting due to change in carer support that resulted in transport difficulty. This person was randomized to control first and had completed initial clinical assessment, but not any subsequent measures or intervention. Of the remaining 38 participants, all completed the 5-week Boot Camp, with 74% completing the full allocation of 90 hours of therapy over 5 weeks. Ten individuals missed treatment sessions due to illness through the program (mean 1.9 ± 0.9; min–max = 1-3 missed sessions). Feedback indicated 87% agreed or strongly agreed that Boot Camp was beneficial and acceptable (Figure 2). There were no hospital admissions, injuries, or treatment-emergent adverse events.

### Clinical Effectiveness

**Figure 3.**
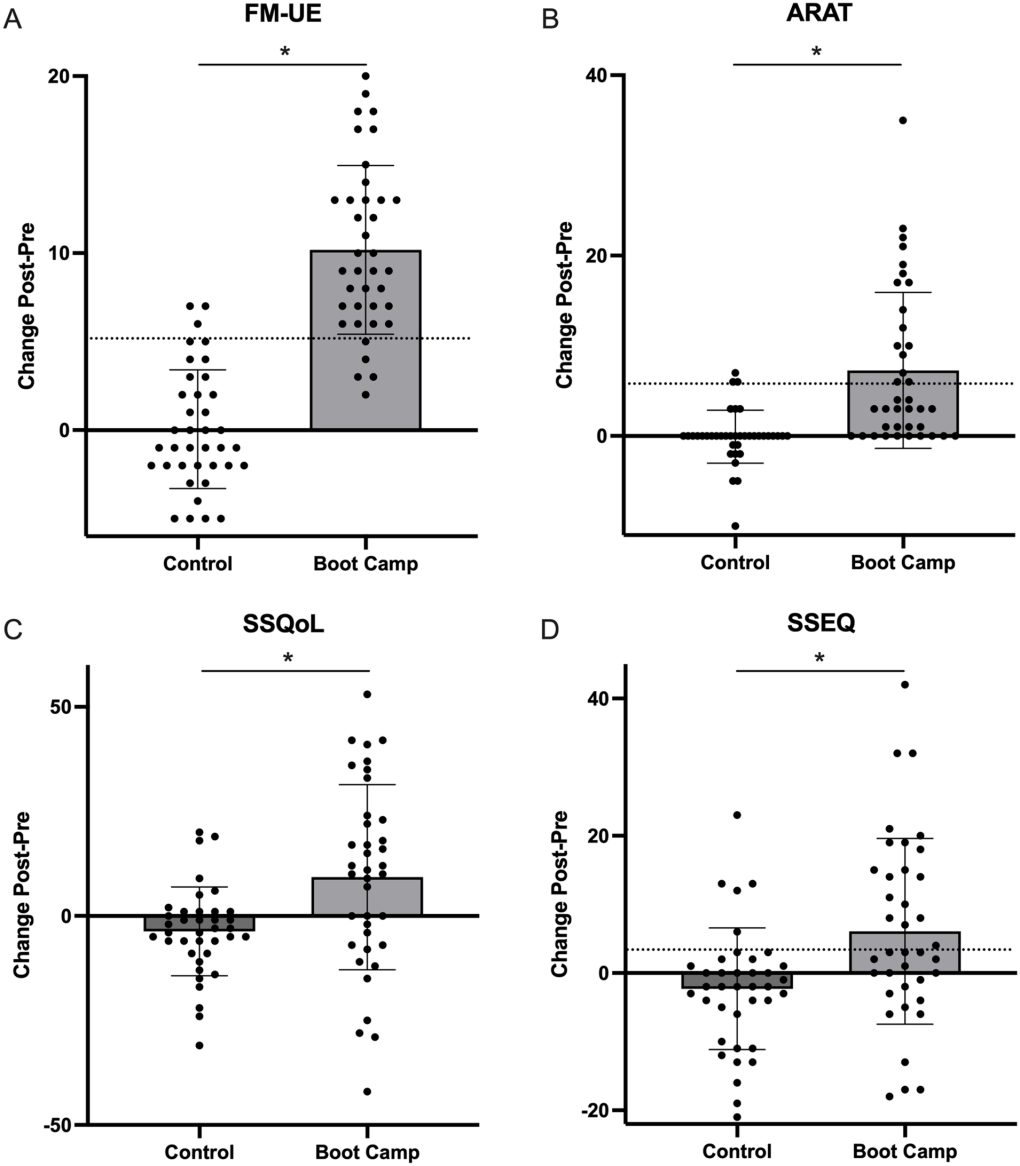
Clinical outcomes from Boot Camp compared to control. (A) Fugl-Meyer Upper Extremity. (B) Action Research Arm Test. (C) Stroke specific quality of life. (D) Stroke Self Efficacy questionnaire. Error bars represent standard deviation, circles represent individual data points. *Statistical significance between Boot Camp and usual care.

Mean pre and post data are reported in Table 2 and full linear mixed model results are in Appendix 3. For FM-UE, there was an effect of Group (β = 7.5, standard error [SE] = 0.7, 95% confidence interval [CI] = 6.1-8.9, *t*_(35)_ = 10.5, *P* < .001) and Group × Timepoint interaction (β = 10.2, SE = 1.0, 95% CI = 8.2-12.1, *t*_(35)_ = 10.5, *P* < .001). FM-UE increased after Boot Camp (mean difference = 10.2, SE = 0.7, 95% CI = 8.6-11.6, *P* < .001), but not control (mean difference = −0.1, SE = 0.5, 95% CI = −1.2 to 1.1, *P* = .92; Figure 3(A)). MEP status was a significant covariate (β = 33.4, SE = 4.5, 95% CI = 24.1-42.7, *t*_(29)_ = 7.4, *P* < .001). Individuals who were MEP+ had higher FM-UE scores prior to Boot Camp (43.4 ± 12.0) than MEP− (13.8 ± 12.2; mean difference = 29.6, SE = 4.3, 95% CI = 21.0-38.3, *P* < .001). However, gain in FM-UE following Boot Camp did not differ between individuals who were MEP+ (∆FM-UE = 10.1 ± 4.4) and MEP− (∆FM-UE = 10.1 ± 4.8; mean difference = 0.0, SE = 1.6, 95% CI = −3.2 to 3.3, *P* = .98). Further exploration of FM-UE subscales found those who were MEP+ had greater gains in coordination (Table 3). Covariates of age, sex, time since stroke, randomization order, side affected were not significant (Appendix 3).

**Table 2.** Clinical Outcomes Pre and Post Both Boot Camp and Control.

	Boot camp		Control	
Measure	Pre (n = 38)	Post (n = 38)	Pre (n = 38)	Post (n = 38)
Fugl-Meyer Upper Extremity	34.1 ± 18.4	44.3 ± 18.2	37.2 ± 19.7	37.3 ± 19.3
Action Research Arm Test	26.0 ± 22.3	33.3 ± 24.7	28.9 ± 23.7	28.8 ± 23.1
Stroke specific quality of life	152.8 ± 41.1	162.1 ± 41.1	158.2 ± 38.8	154.5 ± 38.7
Stroke self-efficacy	85.2 ± 29.0	91.3 ± 28.9	89.7 ± 25.0	87.5 ± 26.6
EQ-5D-5L	0.44 ± 0.39	0.50 ± 0.31	0.45 ± 0.29	0.44 ± 0.35
EQ-VAS	66.8 ± 21.1	71.9 ± 16.7	69.7 ± 14.2	66.5 ± 16.5

Abbreviations: EQ-5D-5L, EuroQol-5 Dimension 5 level; EQ-VAS, EuroQol Visual Analog Scale, SD, standard deviation.

Data presented as mean ± SD.

**Table 3.** Change in Fugl-Meyer Upper Extremity and Action Research Arm Test Subscales After Boot Camp, Separated for MEP Status.

Measures	Subscale	Change value		Statistic^a^
		MEP+ (n = 24)	MEP− (n = 12)	
FM-UE	Arm	4.9 ± 2.4	6.0 ± 4.5	*t*_(34)_ = −0.98, 95% CI = −3.5-1.2, *P* = 1.00
	Wrist	1.8 ± 1.9	1.3 ± 2.5	*t*_(34)_ = 0.78, 95% CI = −0.9-2.1, *P* = 1.00
	Hand	1.4 ± 1.8	2.3 ± 2.3	*t*_(34)_ = −1.19, 95% CI = −2.3-0.6 *P* = .96
	Coordination	2.0 ± 1.3	0.6 ± 1.1	*t*_(34)_ = 3.27, 95% CI = 0.5-2.3 *P* = .008*
ARAT	Grasp	2.9 ± 3.2	0.9 ± 2.9	*t*_(24.3)_ = 1.86, 95% CI = −0.2-4.1, *P* = .30
	Grip	1.5 ± 2.6	0.3 ± 0.9	*t*_(31.3)_ = 2.16, 95% CI = 0.1-2.4, *P* = .15
	Pinch	3.4 ± 4.9	0.6 ± 2.0	*t*_(33.3)_ = 2.43, 95% CI = 0.5-5.1, *P* = .08
	Gross	1.3 ± 1.4	1.7 ± 1.5	*t*_(34)_ = −0.73, 95% CI = −1.4-0.7, *P* = 1.00

Abbreviations: ARAT, Action Research Arm Test; FM-UE, Fugl-Meyer Upper Extremity; MEP, motor evoked potential, SD, standard deviation.

Data presented as mean ± SD.

a Bonferroni adjusted *P*-values.

* Statistically significant difference.

For ARAT, there was an effect of Group (β = 4.4, SE = 1.1, 95% CI = 2.2-6.5, *t*_(35)_ = 4.1, *P* < .001) and Group × Timepoint interaction(β = 7.1, SE = 1.6, 95% CI = 3.9-10.3, t_(35)_ = 4.5, *P* < .001). ARAT scores increased after Boot Camp (mean difference = 7.2, SE = 1.4, 95% CI = 4.3-10.1, *P* < .001), but not control (mean difference = 0.1, SE = 0.5, 95% CI = −0.9 to 1.0, *P* = .91; Figure 3(B). MEP status was a significant covariate (β = 35.2, SE = 5.9, 95% CI = 23.2-47.2, *t*_(29)_ = 6.0, *P* < .001). Individuals who were MEP+ had higher ARAT scores prior to Boot Camp (35.5 ± 19.4) than MEP− (3.4 ± 6.4; mean difference = 32.0, SE = 5.8, 95% CI = 20.3-43.8, *P* < .001). Gain in ARAT following Boot Camp was greater for those MEP+ (∆ARAT = 9.0 ± 9.3) compared to MEP− (∆ARAT = 3.4 ± 5.7), but not significant (mean difference = 5.6, SE = 2.9, 95% CI = −0.3 to 11.6, *P* = .06). Further exploration of ARAT subscales found those who were MEP+ had larger improvements in grasp, grip, and pinch, but significance was lost when correcting for multiple comparisons (Table 3). Covariates of age, sex, time since stroke, randomization order, side affected were not significant (Appendix 3).

There was a Group × Timepoint interaction for SSQoL (β = 12.4, SE = 4.7, 95% CI = 2.9-22.0, *t*_(35)_ = 2.7, *P* = .01). SSQoL increased pre-to-post Boot Camp (mean difference = 8.8, SE = 3.6, 95% CI = 1.4-16.2, *P* = .02), and decreased after control (mean difference = 3.6, SE = 1.8, 95% CI = 0.0-7.2, *P* = .05; Figure 3(C). Covariates were not significant (Appendix 3).

For SSEQ, there was a Group × Timepoint interaction (β = 6.9, SE = 2.9, 95% CI = 1.0-12.9, *t*_(35)_ = 2.4, *P* = .02). SSEQ increased after Boot Camp (mean difference = 4.9, SE = 2.1, 95% CI = 0.7-9.1, *P* = .02), but not control (mean difference = −2.1, SE = 1.5, 95% CI = −5.1 to 1.0, *P* = .17; Figure 3(D)). MEP status was a significant covariate (β = 24.0, SE = 9.2, 95% CI = 5.3-42.8, *t*_(29)_ = −2.6, *P* = .01). Individuals who were MEP+ had higher SSEQ prior to Boot Camp (92.2 ± 27.8) than MEP− (69.8 ± 27.8; mean difference = 22.5, SE = 9.8, 95% CI = 2.5-42.4, *P* = .02). However, gain in FM-UE following Boot Camp did not differ between individuals who were MEP+ (∆SSEQ = 6.5 ± 13.4) and MEP− (∆SSEQ = 1.7 ± 9.8; mean difference = 4.8, SE = 4.4, 95% CI = −4.1 to 13.7, *P* = .28).

Both EQ-5D-5L and EQ-VAS had no effect of Group, Timepoint, or Group × Timepoint interaction (all *P* > .11). EQ-5D-5L increased after Boot Camp (∆EQ-5D-5L = 0.06 ± 0.2) and decreased after control (∆EQ-5D-5L = −0.01 ± 0.2). Time since stroke was a significant co-variate for EQ-5D-5L analysis with longer time associated with reduced EQ-5D-5L (β = −.04, SE = 0.02, 95% CI = −0.08 to 0.00, *t*_(29)_ = −2.2, *P* = .04).

### Sensitivity Analyses

Twenty-six percent of participants missed between 1 and 3 sessions of Boot Camp. There was no difference in clinical measures between those completing the full program (n = 28) and those missing sessions (n = 10; all *P* > .17, Appendix 4). There was no association between number of completed Boot Camp sessions and improvement in clinical measures (all *P* > .26).

Re-analysis of linear mixed models for each clinical outcome was performed excluding those allocated usual care second. Significant Group × Timepoint interactions remained for FM-UE, ARAT, and SSQoL (all *P* < .035), but not for SSEQ (*P* = .062; Appendix 4).

### Participant Experience

Seven participants (4 male, aged 57.9 ± 14.1 years, time since stroke 1.9 ± 2.3 years) completed interviews about their Boot Camp experience. No new themes emerged after the sixth and seventh interviewed participant. Feedback is reported in 4 themes; intensity matters, variety works, peer support, and goals are motivating.

### Intensity Matters

Whilst participants had different degrees of deficit, each noted an improvement in their upper-limb. Boot Camp required a considerable amount of commitment and energy. All interviewed participants noted they did not do any other therapy during this time and rested enough so they were able to commit to the daily sessions.

*In the end, I quite liked the intensity of it. . . Like it was very regimented and had a routine, which I liked.* (Participant (P)10)

*Right from the outset, I would say, be prepared to be exhausted, be prepared to rest. It would be disastrous - if I had been coming out of there, going home and doing 1000 things try to put in a normal day, that would have been. . . self defeating. Instead, I would come home, and I have just enough time to eat lunch, small lunch. And I’d say “See you later” and go and lie down there. And I probably sleep three hours. And it was totally necessary. But if I hadn’t been prepared for that, or if there’d been any input that suggested that I shouldn’t be doing that, everything would have fallen apart.* (P1)

### Variety Works

Different physiotherapists led specific days of the Boot Camp. Participants noted they enjoyed the variety each therapist brought with their experience.

*I liked the fact that there were different people doing it each day, and you got a different perspective. Or you learned something different from everyone, and that their approach was different. And I really enjoyed that.* (P33)

The variety of exercises, not only for task-oriented training (eg, stringing beads, pouring water, and pegs on clothesline), but also technologies such as virtual reality, computer-based games, and assistive exercise devices (like Motomed) offered a good range for participants to feel challenged. They did not find themselves bored due to the amount of different exercises they could do.

### Peer Support

All but 1 participant enjoyed the group format session. That person had higher needs and didn’t feel they were given the therapy they needed, commenting that a one-on-one would have been better for them. All other participants loved the social aspect as many had never spoken with other stroke survivors before these sessions. They were able to talk about their experiences with each other, feeling less alone and isolated, and they had a common lived experience.

*I think the group thing for the Boot Camp was an excellent idea. Because I’m as interested in the other people as I am in anything else. And in fact, I make it a priority interviewing them. I think I’d even use that formal term because I will just go after them because I have things to learn from them.* (P1)

One participant’s wife contacted the clinic after her husband had spent most of the morning speaking with other stroke survivors but one more closely to the deficits he had experienced. It gave him hope and in turn he had become more positive. This hadn’t happened since the stroke (18 months prior; P9).

The exercises were challenging, but being in a social environment allowed them to have more fun concentrating on the tasks, and in some cases, trying to beat each other’s scores.

*Yeah, but it just seemed like you hit the five-week mark, and everyone was starting to really improve, you were seeing improvements. And it would have been nice to take it further.* (P33)

In another instance peer contact needed to be carefully managed by staff. One participant commented:

*. . . there’s potential for someone to come away from it feeling quite disappointed in their . . . lack of abilities. Yeah. Because comparing different, I think similar stroke.* (P10)

### Personalized Attention

Goals were written on the walls in the clinic so that the participants could strive toward them. This brought about more social support and comradery.

*But mostly, I think it was just the attention just to my upper-limb, which I hadn’t experienced before.* (P19)

## Discussion

This study evaluated feasibility, safety, and effectiveness of Boot Camp, a pragmatic, intensive, group-based, 5-week upper-limb rehabilitation program for individuals with chronic stroke. Thirty-nine individuals were randomized, with 38 completing the trial. Feasibility criteria for recruitment, program completion, acceptability, and safety were met, but not adherence to full therapy amount. Boot Camp led to significant, meaningful, short-term improvement in upper-limb motor impairment, activity, quality of life, and self-efficacy compared to the control. Participants reported key themes around intensity, variety, peer support, goals, and motivation. Intensive rehabilitation appears an attractive solution for meaningful improvement after stroke.

Consent from eligible participants (97%) and completion of the 5-week program (100%) were notably high. For reference, a review of 512 stroke rehabilitation trials reported a median recruitment rate of 34%, increasing to 48% for community trials, with 6% drop-out.^35^ We speculate our recruitment and completion rates were high as this was a desired intervention that would otherwise be difficult to obtain in the community. In support, 87% of participants agreed, or strongly agreed, that Boot Camp was acceptable and beneficial. However, the requirement for daily rehabilitation was challenging, with only 74% of participants completing the full therapy duration of 18 hours per week (total 90 hours over 5 weeks). Several participants missed a session due to illness, and additional contact precautions were in place within the clinic during data collection in 2022 due to the COVID-19 pandemic. While program adherence fell below target, it is noteworthy that relatively few sessions were missed (min–max = 1-3, of 25 sessions), appearing to have no significant impact on clinical effectiveness.

Boot Camp led to significant, meaningful, short-term improvement in upper-limb impairment and activity. Gains in both FM-UE (10.2 points) and ARAT (7.3 points) exceeded usual care and MCID thresholds.^16,31^ Participants interviewed about their Boot Camp experience all noted improvement in their paretic upper-limb. The magnitude of clinical gain is similar to previous chronic stroke studies, with FM-UE improvement of 6 to 11 points, and ARAT improvement of 6 points.^15,17^ In our data, it is noteworthy that co-variates of time since stroke and age did not significantly influence the results. Similarly, MEP status, a neurophysiological biomarker of corticospinal tract integrity, had less bearing on our results than anticipated. Although gain in upper-limb activity (ARAT) was greater for those who were MEP+, this was not statistically significant when correcting for multiple comparisons, and both MEP+ and MEP− ARAT scores improved on average. Similarly, gain in upper-limb impairment, assessed by FM-UE, did not differ between those who were MEP+ and MEP−. Our findings are similar to previous work that determined corticospinal tract integrity correlated with motor outcomes in chronic stroke, but not treatment-induced improvement.^36^ However, closer examination points toward a more nuanced truth. FM-UE subscales revealed more proximal upper-limb improvement, although not significant, for MEP− participants, and greater coordination gains for MEP+ participants. This might reflect assessment of MEP status, recorded from electromyography of the distal hand, along with the corticospinal tract having a more pronounced role in the hand,^37,38^ as opposed to the proximal upper-limb where other pathways might contribute.^39,40^ Our interpretation is that intensive rehabilitation for individuals with chronic stroke leads to significant and meaningful gains in upper-limb clinical outcomes irrespective of age, time since stroke, or functional integrity of the corticospinal tract.

The magnitude of improvement in individuals with chronic stroke is somewhat remarkable. Noting participants in the study had stroke 6 months to 17 years prior, our findings refute the notion of a recovery plateau within months of stroke. Behavioral data showing most recovery happens early after stroke has led to a presumption that little improvement beyond 6 months of stroke remains possible.^41-45^ In support, preclinical and human data point to a time-limited period of heightened plasticity within days to weeks of stroke.^8,46^ We argue opportunity for achieving meaningful gains is not limited to a critical window of heightened plasticity. While such a period likely exists, it appears to support accelerated recovery early after stroke. Rather, the recovery plateau likely reflects diminishing therapy as people move out of formal rehabilitation. Our data, and others,^15,17^ clearly show a recovery plateau is not inevitable. Improvement remains possible if afforded appropriate access, amount, and intensity of rehabilitation.

Boot Camp benefits extend beyond upper-limb improvements. There was evidence of improved quality of life and self-efficacy after participating in 5 weeks of intensive therapy. Increased self-efficacy exceeded MCID thresholds,^32^ but gains in quality of life, measured by EQ-5D-5L, fell short of MCID for stroke (0.08-0.13).^47,48^ These findings are supported by qualitative data highlighting that Boot Camp gave hope and enabled people to be more positive about their recovery journey. Improved quality of life and self-efficacy might have occurred for 2 reasons. First, reduced motor impairment can increase participation and activity, boosting quality of life,^49-51^ although others suggest this may have less influence.^52,53^ Second, we should not discount the “group effect” of Boot Camp. Qualitative data emphasized the importance of the social environment and benefits of speaking with other individuals with stroke. Peer support and sharing of experiences are noted benefits of group self-management interventions for stroke.^54^ Intensive rehabilitation in a group setting for individuals with chronic stroke appears an attractive solution, offering resource efficiencies, along with psychological and social benefits.

### Limitations

Perhaps most noteworthy, the outcome assessor was not blind in this study due to funding limitations, and this remains a potential source of bias. While many outcomes were self-reported, and upper-limb gains were supported by participant feedback, highlighting perceived benefit, it is unquestionable that rigor would be improved with blinded outcome assessors in future trials. Second, the cross-over design was selected for this early evaluation to increase efficacy, recruitment, and ensure all participants were afforded the opportunity for Boot Camp participation. As rehabilitation should result in long-term motor learning, a “wash-out” period is likely ineffective between study arms. It is possible carry-over gains from individuals allocated to Boot Camp first could lead to a ceiling effect, limiting potential to show improvement during usual care and inflating the effect of Boot Camp. To account for this, statistical models were adjusted for session order, which did not prove a significant co-variate. Furthermore, a sensitivity analysis excluding those allocated usual care second, demonstrated upper-limb gains remained. Few participants (n = 2) allocated to usual care second had a FM-UE >60, indicating the majority still had capacity to demonstrate meaningful clinical improvement. Furthermore, this trial did not document long-term clinical outcomes. There would be value in knowing whether clinical benefit extends beyond completion of the intervention. Finally, a-priori thresholds for full protocol adherence were not met due to participant illness. While this did not significantly limit gain in clinical outcomes, maintaining protocol adherence is a challenge facing lengthy, demanding, rehabilitation schedules. Solutions to compensate for missed therapy might be required and could include additional “make up” sessions or allowance for telehealth when unwell.

## Conclusion

Boot Camp, a pragmatic, intensive, group-based, 5-week upper-limb rehabilitation program was feasible, safe, and led to large and meaningful gains in upper-limb outcomes in individuals with chronic stroke. Benefits were noted for all individuals, irrespective of severity of arm impairment, stage of recovery, age, or integrity of the corticospinal tract integrity. Stroke services can now consider implementing similar programs with confidence.

## Supplemental Material

sj-docx-1-nnr-10.1177_15459683251348199 – Supplemental material for Boot Camp: A Randomized Cross-Over Trial of Intensive Upper-Limb Rehabilitation After Chronic Stroke Supplemental material, sj-docx-1-nnr-10.1177_15459683251348199 for Boot Camp: A Randomized Cross-Over Trial of Intensive Upper-Limb Rehabilitation After Chronic Stroke by Brenton Hordacre, Jeric Uy, Saran Chamberlain, Ines Serrada, Ashraf N. H. Gerges and Susan Hillier in Neurorehabilitation and Neural Repair
