# Supplementary material for: Boot Camp: A Randomized Cross-Over Trial of Intensive Upper-Limb Rehabilitation After Chronic Stroke
Source: Neurorehabil Neural Repair. 2025 Jul 8;39(10):789–800. doi: 10.1177/15459683251348199 (PMC12476464; doi:10.1177/15459683251348199)
Supplement: sj-docx-1-nnr-10.1177_15459683251348199 – Supplemental material for Boot Camp: A Randomized Cross-Over Trial of Intensive Upper-Limb Rehabilitation After Chronic Stroke [file sj-docx-1-nnr-10.1177_15459683251348199.docx]

**Appendix 1: Qualitative Interview Script**

*Interview Guide*

Please note that this interview is being recorded and will be transcribed for analysis. Do you consent to this?

**Qu 1:** Firstly, please tell me about your experience with the intensive arm recovery program?

**Qu 2**: Describe to me the things you did and didn’t like about this program?

**Qu 3:** Can you tell me about your arm and how it was affected by stroke?

**Qu 4**: Can you tell me about how your participation in activities has changed since your stroke?

**Qu 5**: Can you tell me about how your quality of life has changed since your stroke?

**Qu 6:** Can you tell me about any changes you have noticed in your arm after participating in this program?

**Qu 7:** Would you like to see this program continue in the future?

**Qu 8:** Can you tell me about any changes to the program you would like to see?

**Qu 9:** What else would you like to share about the program that you haven’t had a chance to yet?

**Appendix 2: De-identified Individual Data**

| **ID** | **Age (yrs)** | **Sex** | **Time since stroke (yrs)** | **Allocation (initial arm)** | **Side*** | **MEP status** | **Usual Care** | | | |
| --- | --- | --- | --- | --- | --- | --- | --- | --- | --- | --- |
|  |  |  |  |  |  |  | **Weekly Sessions** | **Duration (min/session)** | **Total weekly therapy (min)** | **Content** |
| 1 | 82.0 | M | 0.9 | Active | L | - | 3 | 45 | 135 | UL and LL mob, bal |
| 2 | 47.8 | F | 9.5 | Active | L | - | 1 | 45 | 45 | UL and LL mob, bal |
| 3 | 62.7 | M | 0.6 | Control | R | + | 2 | 45 | 90 | Fine motor, coordination, balance |
| 4 | 49.2 | F | 5.1 | Control | R | - | NR | NR | NR | NR |
| 5 | 72.2 | M | 0.7 | Control | R | + | 1 | 30 | 30 | UL rehab, fine motor, strength |
| 6 | 42.9 | M | 5.1 | Control | L | + | 1 | 45 | 45 | UL and LL rehab |
| 7 | 54.3 | F | 1.7 | Control | L | + | 1 | 45 | 45 | UL and LL rehab |
| 8 | 70.0 | M | 7.0 | Active | L | - | 1 | 30 | 30 | Balance training |
| 9 | 44.8 | M | 2.5 | Active | L | + | 2 | 45 | 90 | UL and LL rehab |
| 10 | 45.7 | F | 0.9 | Active | L | - | 2 | 60 | 120 | UL and LL rehab |
| 11 | 73.7 | M | 1.7 | Control | L | - | 0 | 0 | 0 | nil |
| 12 | 63.4 | M | 3.3 | Active | L | + | 2 | 45 | 90 | mobility |
| 13 | 60.9 | F | 1.8 | Control | R | - | 1 | 30 | 30 | mobility |
| 14 | 21.9 | F | 0.7 | Control | L | + | 2 | 60 | 120 | UL and LL rehab |
| 15 | 67.3 | F | 7.0 | Control | R | X | 0.5 | 30 | 15 | mobility and balance |
| 16 | 73.1 | M | 6.4 | Active | R | + | 1 | 45 | 45 | UL and LL rehab |
| 17 | 51.7 | M | 1.2 | Control | L | + | 0.5 | 45 | 22.5 | UL and LL rehab |
| 18 | 75.0 | M | 6.0 | Active | R | - | 1 | 45 | 45 | UL rehab, fine motor, strength |
| 19 | 46.7 | F | 0.5 | Active | R | + | 2 | 60 | 120 | UL and LL rehab |
| 20 | 78.6 | M | 2.3 | Active | R | - | 2 | 60 | 120 | UL and LL rehab |
| 21 | 62.5 | M | 0.9 | Control | L | + | 1 | 45 | 45 | UL and LL rehab |
| 22 | 30.5 | F | 17.5 | Active | L | - | 1 | 45 | 45 | UL rehab, fine motor, strength |
| 23 | 71.1 | F | 0.6 | Active | L | X | 0 | 0 | 0 | nil |
| 24 | 63.7 | M | 0.7 | Active | L | + | 1 | 45 | 45 | seated balance, UL rehab |
| 25 | 73.9 | F | 0.7 | Control | L | - | 0 | 0 | 0 | nil |
| 26 | 66.8 | F | 0.5 | Control | R | + | 1 | 45 | 45 | UL rehab, fine motor, strength |
| 27 | 68.5 | F | 0.5 | Control | R | + | 0 | 0 | 0 | nil |
| 28 | 70.2 | M | 3.6 | Control | L | - | 1 | 30 | 30 | UL and LL rehab |
| 29 | 50.0 | M | 2.4 | Active | L | + | 2 | 60 | 120 | UL and LL rehab |
| 30 | 36.8 | M | 1.7 | Control | L | + | 1 | 45 | 45 | UL rehab, fine motor, strength |
| 31 | 72.8 | F | 0.5 | Active | R | + | 2 | 45 | 90 | UL rehab, fine motor, strength |
| 32 | 61.8 | M | 1.9 | Active | L | + | 1 | 45 | 45 | UL rehab, fine motor, strength |
| 33 | 57.2 | F | 0.8 | Control | R | + | 1 | 30 | 30 | UL and LL rehab |
| 34 | 86.5 | M | 4.1 | Active | L | + | 2 | 90 | 180 | UL and LL rehab |
| 35 | 45.0 | M | 0.6 | Active | L | + | 1 | 30 | 30 | UL and LL rehab |
| 36 | 80.9 | M | 3.3 | Control | L | + | 3 | 20 | 60 | UL and LL rehab |
| 37 | 74.7 | M | 4.8 | Active | R | - | 1 | 30 | 30 | LL rehab |
| 38 | 63.7 | F | 0.5 | Control | L | + | 2 | 45 | 90 | UL and LL rehab |
| 39 | 67.6 | F | 0.6 | Active | R | + | 1 | 45 | 45 | UL and LL rehab |

*Side refers to the paretic limb.

F, female; L, left; LL, lower-limb; M, male; NR, not recorded; R, right; UL, upper-limb

+, presence of a motor evoked potential with transcranial magnetic stimulation

-, absence of a motor evoked potential with transcranial magnetic stimulation

X, unable to test motor evoked potentials with transcranial magnetic stimulation due to contraindications

| **ID** | **FM-UE** | | | | **ARAT** | | | | **SSQoL** | | | | **SSEQ** | | | | **EQ-5D-5L** | | | | **EQ-VAS** | | | |
| --- | --- | --- | --- | --- | --- | --- | --- | --- | --- | --- | --- | --- | --- | --- | --- | --- | --- | --- | --- | --- | --- | --- | --- | --- |
|  | Intervention | | Control | | Intervention | | Control | | Intervention | | Control | | Intervention | | Control | | Intervention | | Control | | Intervention | | Control | |
|  | Pre | Post | Pre | Post | Pre | Post | Pre | Post | Pre | Post | Pre | Post | Pre | Post | Pre | Post | Pre | Post | Pre | Post | Pre | Post | Pre | Post |
| 1 | 9 | 26 | 16 | 15 | 56 | 3 | 2 | 2 | 202 | 191 | 190 | 185 | 107 | 89 | 98 | 96 | 0.72 | 0.64 | 0.72 | 0.64 | 100 | 85 | 90 | 93 |
| 2 | 34 | 47 | 42 | 40 | 26 | 4 | 4 | 4 | 212 | 219 | 218 | 212 | 95 | 89 | 90 | 92 | 0.41 | 0.66 | 0.51 | 0.73 | 85 | 75 | 75 | 80 |
| 3 | 54 | 66 | 55 | 54 | 4 | 57 | 56 | 56 | 162 | 120 | 163 | 162 | 101 | 120 | 101 | 101 | 0.81 | 0.74 | 0.49 | 0.81 | 65 | 70 | 60 | 65 |
| 4 | - | - | 22 | - | - | - | 3 | - | - | - | 132 | - | - | - | 42 | - | - | - | -0.02 | - | - | - | 90 | - |
| 5 | 38 | 44 | 33 | 38 | 0 | 43 | 23 | 26 | 222 | 220 | 213 | 222 | 120 | 116 | 108 | 120 | 0.81 | 0.81 | 0.91 | 0.81 | 40 | 85 | 70 | 40 |
| 6 | 31 | 40 | 30 | 31 | 15 | 5 | 4 | 4 | 103 | 75 | 127 | 103 | 42 | 25 | 63 | 42 | -0.31 | -0.31 | 0.03 | -0.31 | 45 | 25 | 60 | 45 |
| 7 | 6 | 10 | 11 | 6 | 3 | 4 | 0 | 0 | 145 | 145 | 146 | 145 | 55 | 55 | 88 | 75 | 0.50 | 0.50 | 0.38 | 0.50 | 60 | 60 | 95 | 60 |
| 8 | 40 | 53 | 51 | 49 | 0 | 50 | 42 | 40 | 154 | 171 | 158 | 157 | 44 | 62 | 49 | 47 | 0.04 | -0.14 | -0.14 | -0.14 | 35 | 30 | 30 | 30 |
| 9 | 24 | 31 | 22 | 24 | 57 | 3 | 3 | 3 | 79 | 97 | 80 | 81 | 28 | 28 | 28 | 28 | 0.00 | 0.06 | 0.00 | 0.00 | 75 | 40 | 60 | 55 |
| 10 | 7 | 20 | 17 | 14 | 0 | 3 | 3 | 3 | 177 | 152 | 152 | 147 | 56 | 57 | 56 | 54 | 0.47 | 0.30 | 0.29 | 0.29 | 95 | 50 | 60 | 50 |
| 11 | 53 | 66 | 56 | 53 | 4 | 57 | 57 | 57 | 214 | 202 | 212 | 214 | 118 | 125 | 118 | 118 | 0.59 | 0.92 | 0.59 | 0.59 | 85 | 90 | 80 | 85 |
| 12 | 7 | 15 | 4 | 4 | 57 | 3 | 0 | 0 | 102 | 113 | 102 | 98 | 44 | 47 | 69 | 70 | -0.39 | 0.08 | 0.35 | -0.26 | 50 | 70 | 60 | 50 |
| 13 | 33 | 41 | 26 | 33 | 3 | 11 | 4 | 4 | 110 | 125 | 114 | 110 | 37 | 45 | 49 | 37 | -0.20 | 0.18 | 0.24 | -0.20 | 60 | 50 | 45 | 60 |
| 14 | 59 | 62 | 55 | 59 | 33 | 57 | 57 | 57 | 197 | 239 | 214 | 197 | 113 | 127 | 115 | 113 | 0.81 | 1.00 | 0.88 | 0.81 | 90 | 97 | 95 | 90 |
| 15 | 17 | 35 | 12 | 17 | 23 | 4 | 0 | 3 | 127 | 149 | 140 | 127 | 66 | 85 | 79 | 66 | 0.39 | 0.50 | 0.39 | 0.39 | 75 | 80 | 75 | 75 |
| 16 | 40 | 55 | 50 | 48 | 44 | 55 | 47 | 42 | 148 | 171 | 160 | 151 | 75 | 96 | 82 | 71 | 0.40 | 0.64 | 0.63 | 0.40 | 90 | 70 | 75 | 70 |
| 17 | 38 | 47 | 32 | 34 | 3 | 44 | 23 | 23 | 145 | 187 | 140 | 145 | 97 | 117 | 108 | 97 | 0.48 | 0.59 | 0.44 | 0.48 | 80 | 85 | 60 | 80 |
| 18 | 31 | 45 | 36 | 34 | 46 | 56 | 48 | 47 | 133 | 169 | 145 | 142 | 101 | 98 | 97 | 91 | 0.39 | 0.57 | 0.57 | 0.39 | 60 | 50 | 55 | 50 |
| 19 | 18 | 36 | 33 | 31 | 3 | 6 | 5 | 5 | 168 | 192 | 189 | 187 | 105 | 104 | 105 | 104 | 0.48 | 0.71 | 0.71 | 0.64 | 30 | 70 | 65 | 55 |
| 20 | 48 | 50 | 48 | 48 | 39 | 55 | 50 | 48 | 87 | 140 | 129 | 114 | 84 | 86 | 86 | 82 | 0.38 | 0.40 | 0.40 | 0.38 | 60 | 70 | 70 | 65 |
| 21 | 12 | 18 | 8 | 12 | 11 | 3 | 3 | 3 | 159 | 169 | 158 | 159 | 90 | 93 | 88 | 90 | 0.58 | 0.60 | 0.59 | 0.58 | 70 | 69 | 57 | 70 |
| 22 | 32 | 52 | 58 | 58 | 0 | 57 | 52 | 47 | 170 | 162 | 155 | 161 | 75 | 117 | 108 | 98 | 0.51 | 0.20 | 0.34 | 0.20 | 50 | 65 | 60 | 55 |
| 23 | 33 | 52 | 52 | 47 | 57 | 25 | 25 | 24 | 181 | 191 | 191 | 180 | 92 | 107 | 107 | 110 | 0.81 | 0.81 | 0.81 | 0.93 | 75 | 85 | 85 | 70 |
| 24 | 4 | 7 | 6 | 5 | 47 | 3 | 3 | 3 | 85 | 102 | 100 | 91 | 37 | 52 | 47 | 44 | 0.05 | 0.20 | 0.06 | -0.02 | 1 | 85 | 70 | 60 |
| 25 | 60 | 65 | 57 | 60 | 3 | 57 | 57 | 57 | 230 | 242 | 236 | 230 | 127 | 130 | 126 | 127 | 1.00 | 1.00 | 0.81 | 1.00 | 100 | 95 | 70 | 100 |
| 26 | 41 | 58 | 34 | 41 | 3 | 57 | 36 | 42 | 159 | 152 | 158 | 159 | 95 | 95 | 89 | 95 | 0.63 | 0.66 | 0.28 | 0.63 | 73 | 75 | 80 | 73 |
| 27 | 4 | 17 | 8 | 4 | 35 | 3 | 3 | 3 | 170 | 155 | 151 | 170 | 66 | 61 | 53 | 66 | 0.22 | 0.45 | -0.12 | 0.22 | 95 | 65 | 55 | 95 |
| 28 | 15 | 22 | 12 | 15 | 51 | 3 | 3 | 3 | 159 | 194 | 181 | 159 | 66 | 98 | 82 | 66 | 0.63 | 0.26 | 0.06 | 0.63 | 70 | 75 | 75 | 70 |
| 29 | 45 | 55 | 55 | 50 | 38 | 41 | 41 | 44 | 156 | 193 | 193 | 179 | 101 | 115 | 115 | 113 | 0.72 | 0.66 | 0.66 | 0.81 | 80 | 80 | 80 | 85 |
| 30 | 48 | 56 | 54 | 53 | 57 | 54 | 54 | 52 | 173 | 169 | 171 | 168 | 114 | 116 | 116 | 112 | 0.60 | 0.79 | 0.79 | 0.60 | 80 | 80 | 80 | 80 |
| 31 | 59 | 66 | 62 | 63 | 53 | 57 | 55 | 55 | 123 | 123 | 121 | 122 | 120 | 124 | 121 | 124 | 0.72 | 0.81 | 0.80 | 0.73 | 80 | 87 | 90 | 85 |
| 32 | 57 | 64 | 62 | 61 | 37 | 57 | 57 | 57 | 147 | 180 | 176 | 171 | 111 | 122 | 120 | 117 | 0.26 | 0.66 | 0.52 | 0.41 | 45 | 85 | 70 | 65 |
| 33 | 47 | 58 | 45 | 47 | 43 | 57 | 47 | 53 | 147 | 163 | 148 | 147 | 101 | 99 | 101 | 101 | 0.79 | 0.54 | 0.79 | 0.79 | 60 | 70 | 60 | 60 |
| 34 | 49 | 55 | 55 | 50 | 0 | 54 | 54 | 44 | 104 | 145 | 145 | 114 | 71 | 103 | 103 | 84 | 0.29 | 0.56 | 0.56 | 0.09 | 60 | 80 | 80 | 40 |
| 35 | 49 | 58 | 58 | 56 | 47 | 49 | 49 | 46 | 108 | 101 | 101 | 119 | 66 | 76 | 76 | 99 | 0.06 | 0.17 | 0.17 | 0.17 | 55 | 65 | 65 | 60 |
| 36 | 2 | 12 | 2 | 2 | 27 | 3 | 0 | 0 | 84 | 96 | 84 | 84 | 57 | 65 | 57 | 57 | 0.05 | 0.01 | 0.05 | 0.05 | 50 | 95 | 50 | 50 |
| 37 | 49 | 58 | 56 | 55 | 57 | 57 | 55 | 55 | 198 | 207 | 201 | 195 | 119 | 113 | 117 | 112 | 0.84 | 0.49 | 0.49 | 0.72 | 80 | 80 | 80 | 80 |
| 38 | 51 | 63 | 45 | 51 | 56 | 50 | 20 | 27 | 166 | 137 | 146 | 166 | 123 | 106 | 90 | 103 | 0.39 | 0.42 | 0.39 | 0.39 | 60 | 70 | 50 | 60 |
| 39 | 53 | 59 | 57 | 55 | 26 | 57 | 57 | 57 | 202 | 202 | 202 | 196 | 119 | 106 | 105 | 101 | 0.81 | 0.81 | 0.81 | 0.81 | 75 | 75 | 75 | 70 |

**Appendix 3: Linear Mixed Model Results**

1. Fugl Meyer Upper Extremity

BIC 875.49

| **Type III Tests of Fixed Effects^a^** | | | | |
| --- | --- | --- | --- | --- |
| Source | Numerator df | Denominator df | F | Sig. |
| Intercept | 1 | 29.008 | 28.736 | <.001 |
| Group | 1 | 35.000 | 9.686 | .004 |
| TimePoint | 1 | 35.000 | 125.907 | <.001 |
| Group * TimePoint | 1 | 35.000 | 111.217 | <.001 |
| Age | 1 | 29.000 | 1.522 | .227 |
| Sex | 1 | 29.000 | .282 | .599 |
| TimeSinceStroke | 1 | 29.000 | 1.629 | .212 |
| Randomisation | 1 | 29.000 | 4.201 | .070 |
| MEPstatus | 1 | 29.000 | 54.195 | <.001 |
| SideAffected | 1 | 29.000 | .094 | .762 |
| a. Dependent Variable: FM-UE. | | | | |

1. Action Research Arm Test

BIC 944.72

| **Type III Tests of Fixed Effects^a^** | | | | |
| --- | --- | --- | --- | --- |
| Source | Numerator df | Denominator df | F | Sig. |
| Intercept | 1 | 29.048 | 18.792 | <.001 |
| Group | 1 | 35.022 | 1.020 | .319 |
| TimePoint | 1 | 35.012 | 24.650 | <.001 |
| Group * TimePoint | 1 | 35.004 | 20.372 | <.001 |
| Age | 1 | 29.031 | .006 | .938 |
| Sex | 1 | 29.031 | .843 | .366 |
| TimeSinceStroke | 1 | 29.031 | 4.080 | .072 |
| Randomisation | 1 | 29.031 | 3.290 | .080 |
| MEPstatus | 1 | 29.031 | 36.090 | <.001 |
| SideAffected | 1 | 29.031 | .437 | .514 |
| a. Dependent Variable: ARAT. | | | | |

1. Stroke Specific Quality of Life

BIC 1199.25

| **Type III Tests of Fixed Effects^a^** | | | | |
| --- | --- | --- | --- | --- |
| Source | Numerator df | Denominator df | F | Sig. |
| Intercept | 1 | 29.020 | 3.446 | .074 |
| Group | 1 | 35.000 | .191 | .665 |
| TimePoint | 1 | 35.000 | 2.510 | .122 |
| Group * TimePoint | 1 | 35.000 | 7.064 | .012 |
| Age | 1 | 29.000 | .467 | .500 |
| Sex | 1 | 29.000 | 1.462 | .236 |
| TimeSinceStroke | 1 | 29.000 | .008 | .929 |
| Randomisation | 1 | 29.000 | .011 | .916 |
| MEPstatus | 1 | 29.000 | 2.194 | .149 |
| SideAffected | 1 | 29.000 | .328 | .571 |
| a. Dependent Variable: SSQoL. | | | | |

1. Stroke Self-Efficacy Questionnaire

BIC 1129.39

| **Type III Tests of Fixed Effects^a^** | | | | |
| --- | --- | --- | --- | --- |
| Source | Numerator df | Denominator df | F | Sig. |
| Intercept | 1 | 29.053 | 7.409 | .011 |
| Group | 1 | 35.002 | .048 | .827 |
| TimePoint | 1 | 35.000 | 1.706 | .200 |
| Group * TimePoint | 1 | 35.000 | 5.654 | .023 |
| Age | 1 | 29.002 | .386 | .539 |
| Sex | 1 | 29.002 | .067 | .798 |
| TimeSinceStroke | 1 | 29.002 | 2.807 | .105 |
| Randomisation | 1 | 29.002 | .251 | .620 |
| MEPstatus | 1 | 29.002 | 6.856 | .014 |
| SideAffected | 1 | 29.002 | .264 | .611 |
| a. Dependent Variable: SSEQ. | | | | |

1. EQ-5D-5L

BIC -21.01

| **Type III Tests of Fixed Effects^a^** | | | | |
| --- | --- | --- | --- | --- |
| Source | Numerator df | Denominator df | F | Sig. |
| Intercept | 1 | 29.137 | 1.068 | .310 |
| Group | 1 | 35.001 | 1.490 | .230 |
| TimePoint | 1 | 35.000 | 2.630 | .114 |
| Group * TimePoint | 1 | 35.004 | 1.307 | .261 |
| Age | 1 | 29.005 | 2.395 | .133 |
| Sex | 1 | 29.005 | .605 | .443 |
| Chronicity | 1 | 29.005 | 4.793 | .037 |
| Randomisation | 1 | 29.005 | .372 | .547 |
| MEPstatus | 1 | 29.005 | 2.408 | .132 |
| AffectedArm | 1 | 29.005 | .203 | .655 |
| a. Dependent Variable: EQ5D. | | | | |

1. EQ-VAS

BIC 1171.00

| **Type III Tests of Fixed Effects^a^** | | | | |
| --- | --- | --- | --- | --- |
| Source | Numerator df | Denominator df | F | Sig. |
| Intercept | 1 | 29.078 | 3.662 | .066 |
| Group | 1 | 35.000 | 1.531 | .224 |
| TimePoint | 1 | 35.000 | .435 | .514 |
| Group * TimePoint | 1 | 35.000 | 1.627 | .210 |
| Age | 1 | 29.001 | 3.533 | .070 |
| Sex | 1 | 29.001 | 1.082 | .307 |
| Chronicity | 1 | 29.001 | 1.531 | .226 |
| Randomisation | 1 | 29.001 | .109 | .744 |
| MEPstatus | 1 | 29.001 | .142 | .709 |
| AffectedArm | 1 | 29.001 | .738 | .397 |
| a. Dependent Variable: EQ5D_VAS. | | | | |

**Appendix 4: Sensitivity analysis results**

| **Measure** | **Change (Post-Pre)** | | | **Statistic** | |
| --- | --- | --- | --- | --- | --- |
|  | **Full Program (n=28)** | **Incomplete Program (n=10)** |  | |  |
| FM-UE | 10.4 ± 5.0 | 9.7 ± 4.1 | t_(36)_ = 0.37, 95%CI; -2.9, 4.3, *p* = 0.71 | |  |
| ARAT | 6.1 ± 7.3 | 10.5 ± 11.5 | t_(36)_ = -1.40, 95%CI; -10.8, 2.0, *p* = 0.17 | |  |
| SSQoL | 11.9 ± 22.2 | 2.0 ± 21.3 | t_(36)_ = 1.22, 95%CI; -6.6, 26.3, *p* = 0.23 | |  |
| SSEQ | 7.7 ± 12.2 | 1.6 ± 16.6 | t_(36)_ = 1.23, 95%CI; -4.0, 16.1, *p* = 0.23 | |  |

ARAT, action research arm test; FM-UE, Fugl-Meyer Upper Extremity; SSEQ, stroke self-efficacy questionnaire; SSQoL, stroke specific quality of life.

**Linear Mixed Models, excluding control data for those allocated to usual care second**

1. Fugl Meyer Upper Extremity – sensitivity analysis

| Type III Tests of Fixed Effects^a^ | | | | |
| --- | --- | --- | --- | --- |
| Source | Numerator df | Denominator df | F | Sig. |
| Intercept | 1 | 29.585 | 29.576 | <.001 |
| Group | 1 | 21.162 | 59.123 | <.001 |
| TimePoint | 1 | 23.846 | 93.568 | <.001 |
| Group * TimePoint | 1 | 21.297 | 57.714 | <.001 |
| Age | 1 | 29.636 | 1.854 | .184 |
| Sex | 1 | 29.310 | .173 | .680 |
| TimeSinceStroke | 1 | 30.094 | 1.886 | .180 |
| Randomisation | 1 | 28.954 | 2.676 | .113 |
| MEPstatus | 1 | 29.471 | 61.962 | <.001 |
| SideAffected | 1 | 29.554 | .106 | .747 |
| a. Dependent Variable: FM-UE. | | | | |

1. Action Research Arm Test – sensitivity analysis

| **Type III Tests of Fixed Effects^a^** | | | | |
| --- | --- | --- | --- | --- |
| Source | Numerator df | Denominator df | F | Sig. |
| Intercept | 1 | 29.096 | 16.037 | <.001 |
| Group | 1 | 29.424 | 21.325 | <.001 |
| TimePoint | 1 | 32.717 | 24.461 | <.001 |
| Group * TimePoint | 1 | 30.793 | 17.997 | <.001 |
| Age | 1 | 29.090 | .007 | .935 |
| Sex | 1 | 29.075 | 1.347 | .255 |
| TimeSinceStroke | 1 | 29.107 | 5.117 | .031 |
| Randomisation | 1 | 29.045 | 1.087 | .306 |
| MEPstatus | 1 | 29.082 | 30.816 | <.001 |
| SideAffected | 1 | 29.087 | .712 | .406 |
| a. Dependent Variable: ARAT. | | | | |

1. Stroke Specific Quality of Life – sensitivity analysis

| Type III Tests of Fixed Effects^a^ | | | | |
| --- | --- | --- | --- | --- |
| Source | Numerator df | Denominator df | F | Sig. |
| Intercept | 1 | 28.895 | 1.865 | .183 |
| Group | 1 | 30.320 | 3.025 | .092 |
| TimePoint | 1 | 30.189 | 2.587 | .118 |
| Group * TimePoint | 1 | 30.834 | 4.875 | .035 |
| Age | 1 | 28.960 | 1.045 | .315 |
| Sex | 1 | 28.435 | 1.827 | .187 |
| TimeSinceStroke | 1 | 29.930 | .156 | .695 |
| Randomisation | 1 | 29.191 | .012 | .915 |
| MEPstatus | 1 | 28.687 | 1.289 | .266 |
| SideAffected | 1 | 28.818 | .464 | .501 |
| a. Dependent Variable: SSQoL. | | | | |

1. Stroke Self-Efficacy Questionnaire – sensitivity analysis

| Type III Tests of Fixed Effects^a^ | | | | |
| --- | --- | --- | --- | --- |
| Source | Numerator df | Denominator df | F | Sig. |
| Intercept | 1 | 22.098 | 11.840 | .002 |
| Group | 1 | 16.627 | .218 | .647 |
| TimePoint | 1 | 25.171 | .704 | .409 |
| Group * TimePoint | 1 | 29.827 | 3.756 | .062 |
| Age | 1 | 20.142 | .353 | .559 |
| Sex | 1 | 17.086 | .014 | .908 |
| TimeSinceStroke | 1 | 27.557 | .830 | .370 |
| Randomisation | 1 | 20.730 | .002 | .969 |
| MEPstatus | 1 | 18.278 | 15.882 | <.001 |
| SideAffected | 1 | 18.084 | 2.643 | .121 |
| a. Dependent Variable: SSEQ. | | | | |
